# Systemic Granulomatous Reaction Secondary to Treatment of Bladder Cancer with Bacillus Calmette-Guerin

**DOI:** 10.4084/MJHID.2012.040

**Published:** 2012-06-18

**Authors:** Caterina Giovanna Valentini, Valentina Bozzoli, Anna Rita Larici, Luigi Maria Larocca, Giovanni Delogu, Giuseppe Leone, Livio Pagano

**Affiliations:** 1 Institute of Hematology, Catholic University, Rome; 2 Department of Bioimaging and Radiological Sciences, Catholic University, Rome; 3 Department of Pathology, Catholic University, Rome; 4 Institute of Microbiology, Catholic University, Rome

## Abstract

Intravesical instillation of Bacillus Calmette-Guérin is the elective treatment for transitional cell and in situ bladder carcinoma. Severe complications occur very seldom, but must be known and promptly recognized. We describe the case of a 48-year-old man, treated with chemo-immunotherapy ten years before for a follicular lymphoma, who developed a systemic granulomatous reaction after his twelfth intravescical BCG instillation for bladder cancer.

## Introduction

Bladder instillations with Bacillus Calmette-Guerin (BCG) are widely used as non-specific effective immunotherapy in the treatment of urothelial carcinoma of the bladder, which accounts for more than 70% of all bladder cancer presentations. Local side effects, usually self-limiting, are frequent and commonly consist of hematuria and dysuria along with cystitis, while adverse systemic reactions, potentially life-threatening, develop in 3 to 5% of the patients and may include fever, pneumonitis, hepatitis, sepsis, disseminated intravascular coagulation, and multi-organ failure.^1^,^2^

## Case report

In February 2010 a 48-year-old man was admitted to our hospital for fever and jaundice appeared few hours after the twelfth BCG bladder instillation because of urothelial carcinoma of the bladder diagnosed two years earlier. The patient was known to our department for a diagnosis of follicular lymphoma made ten years before, and treated with six cycles of CHOP chemotherapy (cyclophosphamide, doxorubicin, vincristine, prednisone) plus Rituximab (Mabthera^®-^Roche), an anti CD20 antibody.

**Figure 1:**
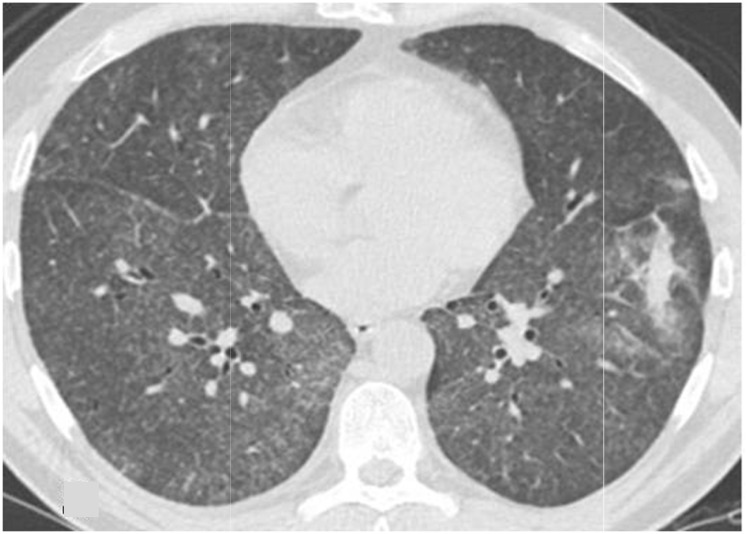
High resolution CT scan of the chest, showing diffuse and bilateral hazy increase of lung density (“ground glass” opacity), a left lower lobe consolidation, multiple fine diffuse nodules bilaterally, suggestive for miliary lung disease.

**Figure 2:**
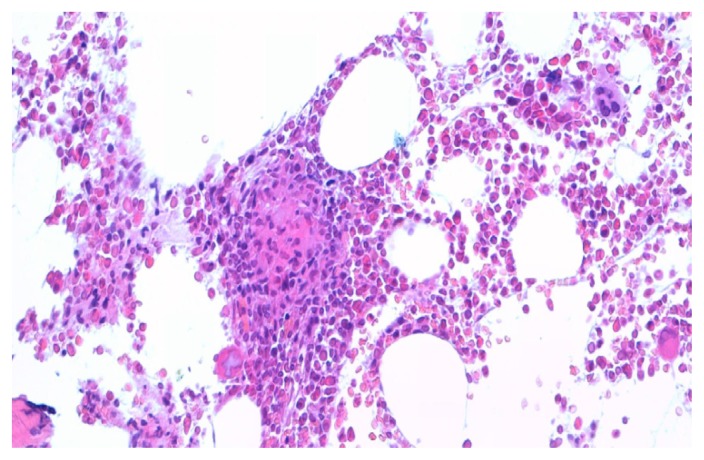
Histological examination of bone marrow biopsy showing epithelioid granuloma (100x).

**Figure 3:**
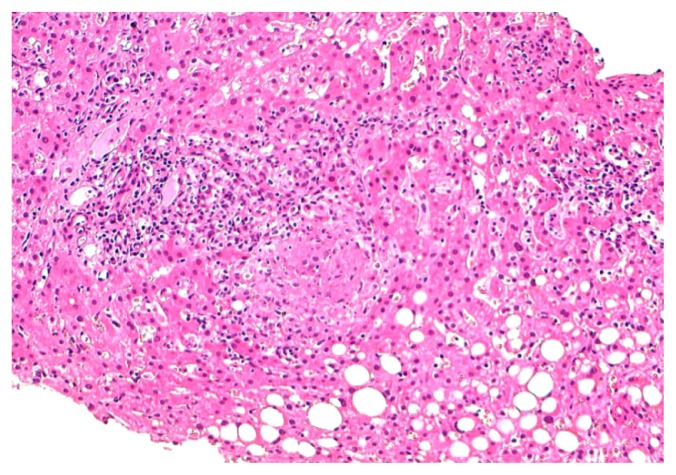
Histological examination of liver biopsy documented non-caseating epithelioid granuloma in the hepatic tissue (100x).

At admission the patient was in fair general conditions but feverish (38,5°C); physical examination revealed scleral and skin jaundice, painful hepatomegaly on palpation, while pulmonary examination was unremarkable and the patient had no peripheral lymphoadenopathy. Complete blood count revealed only a mild hypocromic microcytic anemia with normal platelet and WBC count. Blood chemical assays showed abnormal liver function tests (ALT 265 UI/L, ASL 406 UI/L, serum alkaline phosphatase 785 UI/L, gamma-glutamyl-transferase 370 UI/L, total bilirubin 4,71 mg/dL), elevated LDH (639 UI/L), beta2-microglobulin (6,6 mg/L) and C-reactive protein (92.4 mg/L). Tumor markers were within normal range. Routine blood and urine cultures did not yield any common pathogens and the results of serologic tests in order to assess virus and bacteria exposures were negative. An abdominal ultrasonography confirmed mild hepatomegaly in association with splenomegaly (diameter 13 cm). Chest X-ray showed diffuse bronchial walls thickening and a focal opacity with air bronchogram in the left lower lobe, expression of a possible exacerbation of a concomitant chronic obstructive pulmonary disease. Despite empirical broad-spectrum antibiotic therapy with piperacillin/tazobactam, the patient’s clinical conditions progressively deteriorated and he experienced worsening of fever (40°C), thorax pain and worsening dyspnoea requiring oxygen supply. A high-resolution chest CT-scan was carried out, that confirmed a diffuse and bilateral hazy increase of lung density (“ground glass” opacity), especially in the bases, and the presence of diffuse and bilateral multiple micronodules with random distribution, with thickening of the interlobular septa and a left lower lobe consolidation (Figure 1). A bronchoscopy was carried out and samples of broncho-alveolar lavage were sent for microscopy and culture. To exclude a relapse of his hematological disease, the patient underwent bone marrow trephine biopsy: no evidence of lymphoma relapse was found in his bone marrow specimen, which instead documented multiple aggregates of epithelioid histiocytes setting up for a granulomatous myelitis (Figure 2); finally, because of a continue and gradual increment of liver function tests, particularly of serum alkaline phosphatase (1124 UI/L), and gamma-glutamyl-transferase (481 UI/L), a liver biopsy was performed, whose histological examination documented the presence of several non-caseating epithelioid granulomas with Langhans giant cells revealing a granulomatous hepatitis (Figure 3).

Because of the concomitant radiological findings documented at CT chest scan, suggestive for miliary lung involvement (Figure 1), a systemic infection caused by BCG was suspected on the basis of the recent intravesical administration of BCG and the worsening patient conditions, despite several days of antibiotic treatment. An accurate research of acid-fast bacilli was performed in blood, bone marrow, urine, feces, sputum, biopsy specimens, and broncho-alveolar lavage (BAL) by cultures, PCR analysis and Ziehl-Neelsen staining, with no evidence of *Mycobacterium spp*. Of note a high CD4/CD8 ratio was found in BAL samples (22.73, normal value 1–4); the tuberculin skin test using 2 UI of purified protein derivative (PPD) and the interferon-gamma release assay (Quantiferon) were negative.

So that, although acid-fast bacilli were not elsewhere isolated and blood and bone marrow taken at the same time were PCR negative, ten days after admission an antituberculous treatment was started with isoniazid, rifampicin, and ethambutol, to which ciprofloxacin and corticosteroids were added. After 15 days of antituberculous therapy, fever and dyspnoea subsided and liver-function tests markedly improved along with the patient general well-being. The patient discharged home for the full six-month course of his treatment, at the end of which he was healthy, with complete normalization of inflammation signs and liver function tests, and disappearance of pulmonary nodules and parenchymal abnormalities and of bone marrow granulomatosis, documented at radiologic and histological exams.

## Discussion

BCG is a viable strain of the virulent *Mycobacterium bovis* attenuated through laboratory passage. Local BCG immunotherapy for early stages of urothelial carcinoma is a well established treatment option, and, despite its frequent use, severe systemic complications such as hepatitis, pneumonitis or a septic presentation tend to occur rarely (< 1%). Factors increasing the risk of systemic side effects include bladder biopsy or difficult and traumatic catheterizations of the bladder, situations in which large inoculates of BCG gain easy access to the bloodstream. Use of immunosuppressive agents, diseases such as diabetes, and genetic factors are also important risk factors.^1^

As in the case we described, granulomatous hepatitis can be the leading finding in disseminated BCG infection or can be associated with granulomatous infiltration of other organs, such as lungs and bone marrow.^3^ Bone marrow involvement during disseminated disease with BCG has been reported in only a few cases, where patients showed involvement of the lungs and/or the liver in addition to bone marrow infiltration as part of a septic process,^4^ while miliary tuberculosis represents the most frequent pulmonary complication.^2^,^5^,^6^

In our patient, even without any microbiological evidence of BCG dissemination, we empirically started anti-tuberculous therapy in association with steroids, with a rapid amelioration of symptoms. The pathogenesis of such systemic involvement is controversial; while some authors believe that this is a systemic infection due to hematogenous spread from the bladder, others suppose it is a type IV hypersensitivity mechanism to the BCG, on the basis of the negative Ziehl-Neelsen staining and culture.^4^,^7^,^8^ Moreover, even if the regulation of mycobacterial infections is typically attributed to the CD4+ Th1 cells of the cellular immune response, recent studies suggest that peripheral B cells are important in the host defense against mycobacteria, and a few cases are reported of severe mycobacterial infections in patients receiving B cell depletion therapy.^9^

In our case, diagnosis of hypersensitivity as an additional alternative mechanism was supposed by negative stains and cultures and by the rapid response to steroids, together with an increase in alveolar activated lymphocytes from broncho-alveolar lavage. Furthermore the findings of noncaseating granulomas, the history of BCG administration, and the success of the antituberculous plus steroid therapy support the theory of dissemination of BCG, with subsequent systemic reaction. Noteworthy, for the development of the complications linked to BCG instillations, we can assume that our patient’s susceptibility to the dissemination of *Mycobacterium bovis* could have been influenced by an underlying persistent state of immunosuppression, related to his past medical history of lymphoma, treated with immune-chemotherapy, and to current diagnosis of cancer, generally associated to a various grade of impairment of immune competence.
